# Vaccination and Infection of Swine With *Salmonella* Typhimurium Induces a Systemic and Local Multifunctional CD4^+^ T-Cell Response

**DOI:** 10.3389/fimmu.2020.603089

**Published:** 2021-01-11

**Authors:** Selma Schmidt, Elena L. Sassu, Eleni Vatzia, Alix Pierron, Julia Lagler, Kerstin H. Mair, Maria Stadler, Christian Knecht, Joachim Spergser, Marlies Dolezal, Sven Springer, Tobias Theuß, Vicky Fachinger, Andrea Ladinig, Armin Saalmüller, Wilhelm Gerner

**Affiliations:** ^1^ Institute of Immunology, Department of Pathobiology, University of Veterinary Medicine, Vienna, Austria; ^2^ University Clinic for Swine, Department for Farm Animals and Veterinary Public Health, University of Veterinary Medicine, Vienna, Austria; ^3^ Clinic for Poultry and Fish Medicine, Department for Farm Animals and Veterinary Public Health, University of Veterinary Medicine, Vienna, Austria; ^4^ Institute of Microbiology, Department of Pathobiology, University of Veterinary Medicine, Vienna, Austria; ^5^ Platform for Bioinformatics and Biostatistics, Department of Biomedical Sciences, University of Veterinary Medicine, Vienna, Austria; ^6^ Ceva Innovation Center GmbH, Dessau-Roßlau, Germany

**Keywords:** interferon-γ, interleukin-17A, pig, *Salmonella* Typhimurium, multifunctional T cells, lamina propria lymphocytes, tumor necrosis factor-α

## Abstract

The gram-negative facultative intracellular bacteria *Salmonella* Typhimurium (STM) often leads to subclinical infections in pigs, but can also cause severe enterocolitis in this species. Due to its high zoonotic potential, the pathogen is likewise dangerous for humans. Vaccination with a live attenuated STM strain (Salmoporc) is regarded as an effective method to control STM infections in affected pig herds. However, information on the cellular immune response of swine against STM is still scarce. In this study, we investigated the T-cell immune response in pigs that were vaccinated twice with Salmoporc followed by a challenge infection with a virulent STM strain. Blood- and organ-derived lymphocytes (spleen, tonsils, jejunal and ileocolic lymph nodes, jejunum, ileum) were stimulated *in vitro* with heat-inactivated STM. Subsequently, CD4^+^ T cells present in these cell preparations were analyzed for the production of IFN-γ, TNF-α, and IL-17A by flow cytometry and Boolean gating. Highest frequencies of STM-specific cytokine-producing CD4^+^ T cells were found in lamina propria lymphocytes of jejunum and ileum. Significant differences of the relative abundance of cytokine-producing phenotypes between control group and vaccinated + infected animals were detected in most organs, but dominated in gut and lymph node-residing CD4^+^ T cells. IL-17A producing CD4^+^ T cells dominated in gut and gut-draining lymph nodes, whereas IFN-γ/TNF-α co-producing CD4^+^ T cells were present in all locations. Additionally, the majority of cytokine-producing CD4^+^ T cells had a CD8α^+^CD27^-^ phenotype, indicative of a late effector or effector memory stage of differentiation. In summary, we show that *Salmonella*-specific multifunctional CD4^+^ T cells exist in vaccinated and infected pigs, dominate in the gut and most likely contribute to protective immunity against STM in the pig.

## Introduction

*Salmonella* Typhimurium (STM) is a gram-negative facultative intracellular bacterium that belongs to the family of the *Enterobacteriaceae* and is able to infect a broad range of hosts. Non-typhoidal *Salmonella* serovars such as STM frequently cause food-borne gastroenteritis in humans due to their zoonotic properties and pose a permanent risk for food safety (1, 2). Apart from eggs and egg products, pigs and pork are among the top transmission vectors of STM to humans *via* the food chain (3, 4).

Salmonellosis in the pig can manifest as diarrhea and lethargy in weaned pigs; however, in many cases, pigs are infected subclinically and often become carrier animals with *Salmonella* persisting in tonsils, gut and gut-associated lymphoid tissues (5). In addition to hygiene measures and feed intervention strategies, vaccination is considered an effective tool in controlling *Salmonella* in affected pig farms (6). The live attenuated histidine-adenine auxotrophic vaccine Salmoporc (Ceva Santé Animale, Libourne, France, formerly IDT Biologika GmbH) is commercially available in Europe, and has already been proven to reduce clinical signs, shedding and tissue colonization in pigs in several studies (7–13).

So far, studies covering the immune response against STM in the pig have focused on detecting signs of antibody-mediated immunity. Vaccination of pigs with Salmoporc has been shown to induce *Salmonella*-specific IgM, IgG and IgA antibodies (11, 14). Studies in which pigs were infected with a virulent STM strain observed an increase of *Salmonella*-specific IgM antibodies with IgM-IgG class switch occurring 14 days post infection, as well as the induction of *Salmonella*-specific IgA antibodies (15, 16). In contrast to the humoral immune response, the cell-mediated immune response to STM in swine has not been investigated in detail so far. Existing studies have mainly focused on measuring mRNA expression of cytokines in STM-infected piglets (17–20).

STM infection has been more intensively studied in mice where CD4^+^ T cells were shown to have a dominant role in primary clearance of infection (21, 22). Especially the development of Th1 cells and their production of tumor necrosis factor-α (TNF-α) and interferon-γ (IFN-γ), leading to the activation of macrophages, is required for successful bacterial killing (23–26). Additionally, interleukin-17A (IL-17A) produced by Th17 cells contributes to protection by recruiting neutrophils to the intestine and regulating the expression of epithelial tight junction proteins (27, 28). In fact, *Salmonella*-specific Th1 and Th17 cells have been shown to develop simultaneously in spleen and intestine of orally infected mice (29). More recent studies in mice have also demonstrated the importance of non-circulating tissue-resident memory Th1 cells which are able to react immediately in case of a re-infection (30). In contrast to mice in which STM leads to septicemia, STM colonization in the pig is mainly focused on the intestinal tract. The local cellular immune response mounted in gut and mesenteric lymph nodes as well as the generation of memory T cells within these tissues is therefore of great interest. Although the identification of tissue-resident T cells (Trm) in swine is not yet possible due to a lack of antibodies specific for Trm associated markers, differentiation of CD4^+^ T cells in the pig can be analyzed by the expression of CD8α and CD27 (31). In this way three major subsets within porcine CD4^+^ T helper cells can be identified: the subset of CD8α^-^CD27^+^ CD4^+^ T cells constitutes naïve cells, while the CD8α^+^CD27^+^ and CD8α^+^CD27^-^ subsets represent central and effector memory CD4^+^ T cells, respectively (32).

Due to the lack of knowledge on T-cell mediated immunity in pigs in response to STM infections, we studied the antigen-specific CD4^+^ T-cell immune response of swine, both locally and systemically. CD4^+^ T cells and their production of IFN-γ, TNF-α and IL-17A were analyzed in various organs by intracellular cytokine staining (ICS) after *in vitro* stimulation with *Salmonella* antigen. Our results show the induction of STM-specific multifunctional CD4^+^ T cells after vaccination and infection with STM that predominantly possessed an effector memory phenotype and dominated in the porcine intestine.

## Materials and Methods

### Animal Vaccination and Infection Experiment

**Figure 1:**
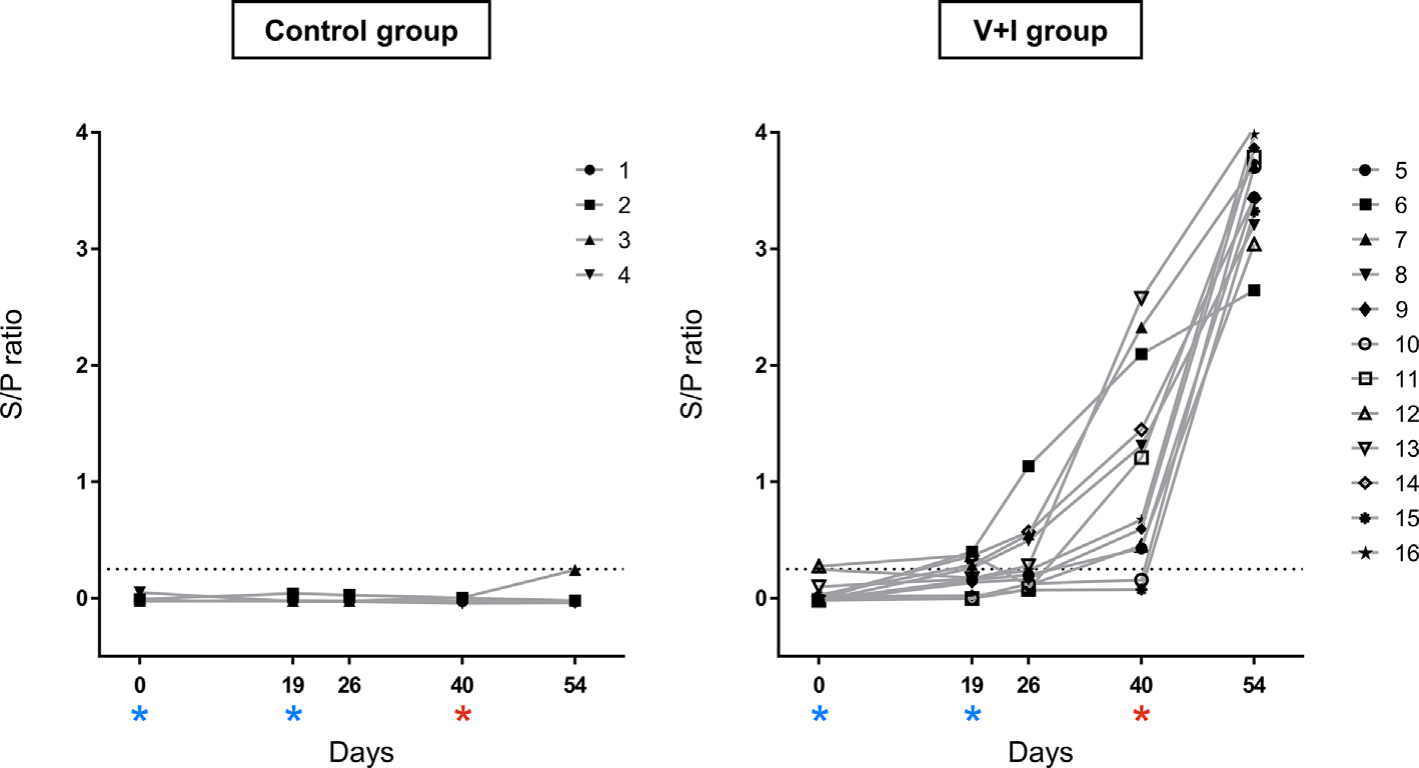
*Salmonella*-specific antibodies in serum over the course of the experiment. *Salmonella*-specific antibodies were measured by ELISA in serum of all animals. Sample to positive (S/P) ratios were calculated by dividing optical densities (OD) of samples by OD of the positive reference control. The dotted line represents the cut-off at a S/P ratio of 0.25. Results < 0.25 were defined as negative, samples ≥ 0.25 as positive. The left graph shows ratios for control animals, the right graph for vaccinated and infected animals. Dates of vaccinations (SD0, SD19) are indicated by blue asterisks, date of infection (SD40) by a red asterisk. The combination of different symbols and numbers represent values of individual animals.

Prior to the study, sows (Large White x Landrace) from a University-owned pig farm in Lower Austria were tested for *Salmonella*-specific antibodies by the IDEXX Swine *Salmonella* Ab Test (IDEXX Europe, Hoofddorp, The Netherlands). The five sows with the lowest sample to positive (S/P) ratios were selected and sixteen four-week-old male castrated pigs (Large White x Landrace x Pietrain) from those sows were included in the study. Piglets at this farm are routinely vaccinated against PCV-2 (Ingelvac CircoFLEX^®^, Boehringer-Ingelheim, Ingelheim am Rhein, Germany) at three weeks of age and *Mycoplasma hyopneumoniae* (M^+^PAC^®^, MSD Animal Health, Kenilworth, USA) in the first and third week of life. After arrival, the animals were weighed and the data was used to achieve a similar distribution of animals with different body weights in two groups: a control group of four animals (pig #1-4) and a group of twelve animals that was later vaccinated and challenge-infected (pig #5-16, V+I). Animals were housed in two separate rooms on straw bedding for the first six weeks. One week before the infection, all animals were moved into a biosafety level (BSL) 2 facility at the University of Veterinary Medicine Vienna with the control group and the V+I group accommodated in separate compartments of the facility. The *Salmonella*-free status of the piglets was validated by serological testing for *Salmonella*-specific antibodies by the IDEXX Swine *Salmonella* Ab Test (IDEXX Europe) (**Figure 1**). Moreover, fecal samples were collected 9, 7 and 6 days prior to the first vaccination and tested for *Salmonella*. The methodology for this microbiological testing is described below in chapter 2.4. All piglets tested negative for *Salmonella* in the feces at the indicated time points.

Starting at five weeks of age, the V+I group was orally vaccinated twice in a 19-day interval [study days (SD) 0 and 19] with 1 ml of the live attenuated histidine-adenine auxotrophic STM vaccine (Salmoporc, Ceva Santé Animale, Dessau-Roßlau, Germany) containing a dose of 1.33 x 10^9^ colony forming units (cfu). The control group received 1 ml of tap water orally. The vaccine was applied using a button cannula attached to a 5 ml syringe. Three weeks after the second immunization (SD40), animals of the V+I group were orally challenged with 5 ml per animal containing 1 x 10^9^ cfu/ml of a virulent monophasic STM strain (DT193, no. RKI 06-1900, described by (33) and provided by Ceva Innovation Center GmbH). For the application *via* an oral drencher kit (Ceva Santé Animale) the challenge strain was mixed with sugar beet solution to enhance acceptance by the animals. The control group received 5 ml of sugar beet syrup diluted in water. Two weeks after infection, necropsy was performed over five consecutive days (SDs 52–56). All animals were anaesthetized by intramuscular injection of Ketaminhydrochlorid (Narketan^®^, 10 mg/kg body weight, Vétoquinol, Lure Cedex, France) and Azaperon (Stresnil^®^, 1.3 mg/kg body weight, Elanco, Greenfield, USA) followed by euthanasia *via* intracardial injection of T61^®^ (tetracaine hydrochloride, mebezonium iodide and embutramide, 1 ml/10 kg body weight, MSD Animal Health). The animal experiment was approved by the institutional ethics committee, the Advisory Committee for Animal Experiments (§12 of Law for Animal experiments, Tierversuchsgesetz - TVG) and the Federal Ministry for Science, Research and Economy (BMWF-68.205/0241-WF/V/3b/2016).

### Clinical Examination, Necropsy and Sample Collection

Clinical monitoring of the animals was performed daily and observations were recorded and evaluated by a scoring system, considering rectal temperature, diarrhea, vomiting and changes in behavior. In addition, body weight of all animals was recorded on a weekly basis. After euthanasia inner organs of all animals were evaluated for pathological changes by visual examination. Fecal samples were taken from all animals at the beginning of the study (SDs −9, −7, −6) as well as in two-week intervals after the first vaccination (SDs 12, 26) for bacteriological examination. Blood samples from the jugular vein (*Vena jugularis*) were taken from all animals prior to vaccination (SD0) and prior to second immunization and challenge infection (SDs 19, 26, 40). On necropsy days (SDs 52–56) blood was drawn by cardiac puncture from anaesthetized animals prior to euthanasia. On these days, samples were also collected from liver, spleen, tonsils, jejunal lymph nodes, ileocolic lymph nodes, jejunum, ileum and cecum. Tissue samples from liver, jejunum, ileum and cecum were always taken from the same area of the organ. The liver sample was taken from the dorsal portion of the left lobe. The jejunum was fully unrolled and the middle determined. From this point, 15 cm of tissue was dissected in the oral and aboral direction (i.e. total length of 30 cm). For the sample of the ileum, a 30 cm section orally of the *Ostium ileale* was collected. A ligation 5 cm from the cecal tip provided the sample of the cecum.

### Detection of S*almonella*-Specific Antibodies in Serum

Serum was obtained after centrifugation of blood samples for 10 min and 1,900 × *g* at room temperature. For the detection of *Salmonella*-specific antibodies, serum samples were tested using a commercially available ELISA kit (IDEXX Swine *Salmonella* Ab Test, IDEXX Europe, Hoofddorp, The Netherlands) according to the manufacturer’s instructions. Results were recorded as S/P ratios determined by the ratio between mean optical density (OD) of each sample and mean OD of the positive control. According to the manufacturer’s recommendations, S/P ratios < 0.25 were defined as negative and samples ≥ 0.25 as positive.

### Microbiological Investigation

Liver, spleen, tonsils, jejunal lymph nodes, ileocolic lymph nodes, jejunum, ileum and cecum from euthanized animals as well as fecal samples taken before and after both immunizations of the animals were investigated for the presence of *Salmonella enterica*. Samples were streaked onto Xylose-Lysine-Deoxycholate (XLD) agar plates (BBL™, Becton Dickinson (BD), Heidelberg, Germany) and incubated in ambient air at 37°C for 48 h. In addition, all samples were pre-enriched in buffered peptone water (BPW, Millipore™, Merck KGaA, Darmstadt, Germany) for 24 h at 37°C. After incubation 0.1 ml of each culture was transferred to Rappaport-Vassiliadis R10 and Selenite broth (both Difco™, BD), incubated for 24 h at 42°C, and subsequently sub-cultured onto XLD agar plates (BBL™, BD) and incubated aerobically at 37°C for 48 h. Presumptive *Salmonella* colonies were confirmed by MALDI TOF mass spectrometry.

### Isolation of Lymphocytes

Peripheral blood mononuclear cells (PBMCs) were isolated from heparinized blood sampled in monovettes (Kabe Labortechnik, Nümbrecht-Elsenroth, Germany) by density gradient centrifugation (Pancoll human, density: 1.077 g/ml, PAN Biotech, Aidenbach, Germany; 30 min at 920 x *g*). Lymphocytes from spleen, tonsil and mesenteric lymph nodes were isolated as described previously (31). To collect lamina propria lymphocytes (LPL) from jejunum and ileum, opened intestines were rinsed in sterile phosphate-buffered saline (PBS, PAN Biotech) and dissected into small pieces (approx. 2x2x2 mm). Subsequently, the tissue was incubated for 60 min at 37°C on a shaker in HBSS supplemented with 2 mM DTT (Carl Roth GmbH+Co.KG, Karlsruhe, Germany), 0.1 mM EDTA (Carl Roth) and 25 U/ml DNase Type 1 (ThermoFisher, Waltham, MA, USA) for the release of intra-epithelial lymphocytes. Supernatants from this incubation were discarded and the remaining tissue was placed in cell culture medium (RPMI 1640 supplemented with 100 IU/ml penicillin and 0.1 mg/ml streptomycin (all PAN Biotech)) to remove traces of DTT and EDTA for 15 min at 37°C. Thereafter, the tissue was transferred to cell culture medium supplemented with 25 U/ml DNase Type 1 and 300 U/ml Collagenase 1 (ThermoFisher) for enzymatic degradation. After incubation for 60 min on a shaker at 37°C, supernatants were collected, centrifuged at 4°C and 600 x *g* for 10 min and the cell pellet was re-suspended and filtered through a cell strainer. Cells were re-suspended in 40% Percoll^®^ (GE Healthcare Bio-Sciences, Pittsburgh, PA, USA) and under-layered with 70% Percoll before being centrifuged at room temperature for 30 min at 920 x *g*. Cells from the interphase were collected, washed twice with PBS and once with cell culture medium (as above but with 5% fetal calf serum (FCS, Merck, Darmstadt, Germany). Afterwards, cells were re-suspended in cell culture medium supplemented with gentamicin and 10% FCS. All cell preparations were counted in a Sysmex XP 300 hematology analyzer (Sysmex Europe GmbH, Norderstedt, Germany).

### Preparation of *Salmonella* Antigen for *In Vitro* Stimulation

Antigens for *in vitro* stimulation of lymphocytes were prepared as follows. The vaccine strain (STM no. 421/125) and the challenge strain (STM no. RKI 06-1900) were cultured *via* two precultures in STM 6/83 medium (in-house) at 37°C and 150 rpm on a shaker. The culture was then centrifuged at 7000 x *g* for 10 min and the pellet re-suspended in PBS. After determination of colony forming units, the concentrate was heat-inactivated in a water bath at 60°C for 90 min. Subsequently, the antigen was aliquoted and stored at −80°C until use.

### *In Vitro* Stimulation and ICS

For intracellular staining of IFN-γ, TNF-α and IL-17A, 5 x 10^5^ freshly isolated cells were cultivated in 200 µl/well of round-bottomed 96-well microtiter plates (Greiner Bio One, Frickenhausen, Germany) For stimulation, wells received either heat-inactivated 1.9 x 10^8^ cfu/ml STM vaccine strain or heat-inactivated 2.7 x 10^8^ cfu/ml STM challenge strain. In pilot experiments, we had established that frequencies of cytokine-producing CD4^+^ T cells plateaued at STM doses >10^8^ cfu/ml (data not shown). The plates were then cultured for approximately 19 h at 37°C. Cells incubated in cell culture medium only served as negative controls. During the last 4 h of culture, Brefeldin A (BD GolgiPlug™, BD Biosciences, San Jose, CA, USA) was present in microcultures at a final concentration of 1 μg/ml. Cultivated cells were harvested and re-suspended in buffer containing PBS with 3% FCS. To analyze the phenotype of lymphocyte subsets by flow cytometry (FCM), cells were surface-stained with primary monoclonal antibodies directed to CD4 (mIgG2b, clone: 74-12-4), CD8α (mIgG2a, clone: 11/295/33, biotinylated) and CD27 (mIgG1, clone: b30c7), all produced and prepared in-house. Binding of primary antibodies was detected by the following secondary reagents: goat anti-mouse IgG2b-A488, rat anti-mouse IgG1-PE-Cy7 (both ThermoFisher) and Streptavidin-BV421 (BioLegend, San Diego, CA, USA). For exclusion of dead cells, Fixable Viability Dye eFlour780 (ThermoFisher) was used according to manufacturer’s protocol with 0.025 μl reactive dye per sample. Free binding sites of secondary antibodies were blocked with whole mouse IgG molecules (2 µg per sample; Jackson ImmunoResearch Laboratories, West Grove, PA, USA). Thereafter, samples were fixed and permeabilized with BD Cytofix/Cytoperm™ Fixation/Permeabilization Kit (BD Biosciences) according to manufacturer’s instructions. This was followed by intracellular staining with IFN-γ-PE (mIgG1, clone: P2G10, BD Biosciences), TNF-α-BV605 (mIgG1, clone: Mab11, BioLegend) and IL-17A-A647 (mIgG1, clone: SCPL1362, BD Biosciences). All incubation steps were performed in 96-well round-bottom plates for 20 min at 4°C with the exception of the intracellular staining step that was carried out for 30 min. FCM analyses were performed on a FACSCanto™ II (BD Biosciences). Data of at least 1 x 10^6^ lymphocytes per sample for PBMC, spleen, tonsils and lymph nodes were recorded. For cells isolated from jejunum and ileum at least 3 x 10^5^ lymphocytes were recorded. Data were analyzed with FlowJo™ Software for Windows (Version 10.4.1; FlowJo, Ashland, OR, USA).

### Statistical Analysis

Data for the graphs in **Figures 1**, **7**, and **S2** including calculation of mean, median and standard deviations were analyzed with GraphPad Prism 5 (GraphPad Software, San Diego, CA, USA). Statistical analysis was performed in R version 3.6.2 (R Core Team (2019). R: A language and environment for statistical computing. R Foundation for Statistical Computing, Vienna, Austria. URL https://www.R-project.org/). We fitted univariate linear mixed models using function *lmer* in R package *lme4* v1.1-21 (34) with log10 transformed frequencies of cytokine-producing CD4^+^ T cells as “response”. Main effects of animal treatment and *in vitro* stimulation and the interaction between them were modeled as fixed categorical effects with two levels (control versus infected) and three levels (medium, vaccine strain and challenge strain), respectively. A random intercept pig effect was included in the model to account for the covariance structure (multiple observations per pig) in our data. For the hypothesis testing, we used maximum likelihood estimation by setting option REML to false. All assumptions for linear mixed models were met. Residuals and random intercepts were normally distributed and residuals homoscedastic. We verified the absence of collinearity *via* generalized variance inflation factors (35) using function *vif* in package *car* v3.0-8 (36). We calculated contrasts between least square means of animal treatment and *in vitro* stimulation levels respectively with package *emmeans* v1.4.7 (37). Significance was declared at a multiple testing corrected 10% false discovery rate (38). We further performed Principal Component Analysis with package *factoextra* v1.0.7 (39). We produced biplots using function *fviz_pca_biplot*, which display PCA scores of samples (shown as dots) and loadings of each variable (shown as vectors) in the same graph. Dots that are close to each other represent samples with similar values. The longer a vector of a variable the bigger the influence of this variable on that principal component. Vectors pointing in a similar direction, forming small angles between them can be interpreted as positively correlated, vectors forming an angle of 90° as uncorrelated and vectors pointing in opposing directions as negatively correlated. We also produced heatmaps with function *heatmap.2* in package *gplots* v3.0.3. (40). Hierarchical clustering using option *method= “ward.D2”*, based on Euclidian distances for rows and columns are shown as dendrograms. For these multivariate descriptive plots, centered and scaled frequencies of cytokine-producing CD4^+^ T cells corrected for individual pig effects were used. We calculated these residuals by subtracting restricted maximum likelihood BLUP animal effects, (option REML set to true), estimated from the same linear mixed model as used for hypothesis testing, from the log10 transformed raw frequencies of cytokine-producing CD4^+^ T cells. During statistical analysis, one animal from the control group (pig #4) was found to be an outlier in the majority of analyzed tissues and was therefore excluded from the ICS data set and subsequent multivariate plots as well as hypothesis testing of cytokine-producing phenotypes for all tissues.

## Results

### Clinical Signs, Serology, and Microbiological Investigation

Due to a lack of knowledge on the T-cell response of pigs against STM, the animal experiment for this study was designed to achieve a strong stimulation of the immune system of pigs in the V+I group by a two-time vaccination and subsequent challenge infection, based on previous observations in demonstrating the efficacy of the Salmoporc vaccine (11). With this study design, no adverse effects on the health of the pigs following infection were expected. Indeed, average daily gain of weight developed homogeneously for both groups for the duration of the study. After moving to the BSL2 animal facility, both groups showed an increase in rectal temperature for one day with temperatures ranging between 39.3°C and 40.4°C and an average increase of 0.35°C in the control group and 0.2°C in the V+I group. This rise in temperature can most likely be attributed to the stress of being moved and adapting to a new environment. After that, all animals showed rectal temperatures within a physiologic range until the end of the study. In addition, mild signs of diarrhea (e.g. pasty feces) were observed in a small number of V+I animals during a three-day interval after challenge infection (data not shown).

To confirm a successful STM infection of the animals, production of serum antibodies as well as STM presence in organs were investigated. The humoral response against STM was evaluated by use of the commercially available IDEXX Swine *Salmonella* Ab Test (**Figure 1**). Results of the *Salmonella* antibody ELISA confirmed the *Salmonella*-free status of the animals at the beginning of the study (SD0). All pigs from the control group remained serologically negative for *Salmonella* sp. throughout the whole course of the experiment. S/P ratios of animals from the V+I group rose moderately after the second immunization and a further substantial increase was observed after challenge infection on SD40.

**Figure 2:**
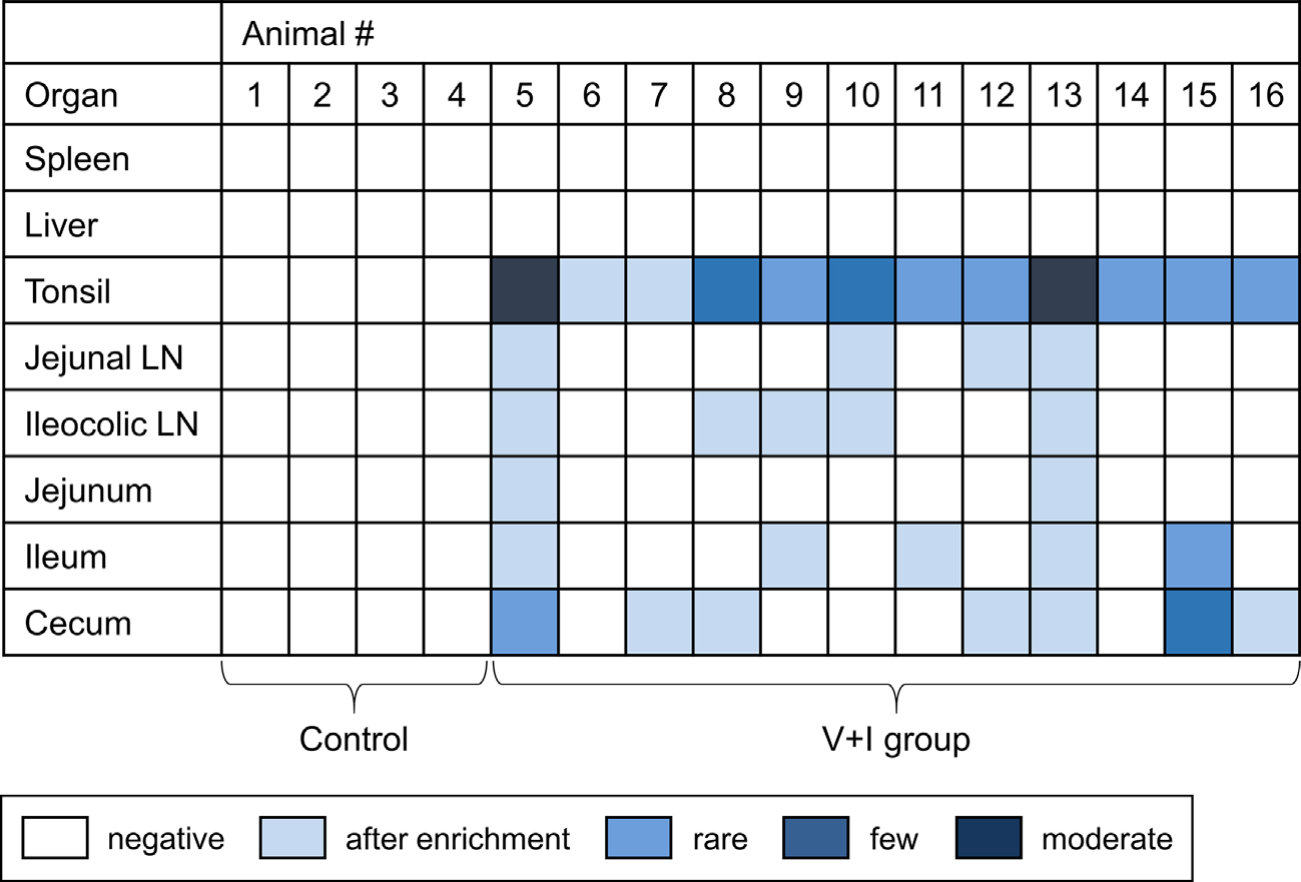
Detection of *Salmonella* Typhimurium on the day of necropsy. Microbiological analysis was performed for spleen, liver, tonsil, jejunal lymph node, ileocolic lymph node, jejunum, ileum and cecum. Results are displayed as a heat map: dark blue/blue boxes indicate detection of *Salmonella* Typhimurium by agar isolation. Light blue boxes indicate isolation after enrichment. White boxes display negative findings for *Salmonella* Typhimurium.

Samples from spleen, liver, tonsils, jejunal lymph nodes, ileocolic lymph nodes, jejunum, ileum and cecum taken at necropsy for each animal were analyzed for the presence of *Salmonella enterica*. No *Salmonella* spp. could be detected in the control group in any of the sampled organs (**Figure 2**). Regarding the V+I group, *Salmonella* spp. could be isolated from all three selected parts of the gut, the jejunal lymph nodes and the ileocolic lymph nodes from at least two animals. Additionally, *Salmonella* spp. were also identified in the tonsils of all V+I animals. Spleen and liver were negative for both control group and V+I group. As the pigs were kept under isolated conditions and the vaccine strain has a limited persistence, it can be assumed that the detection of *Salmonella* spp. is the STM challenge strain. Fecal samples collected prior to (SDs -9, -7, -6) and after both vaccinations (SDs 12, 26) of all animals (control and V+I group) tested negative for *Salmonella* spp. as well (data not shown).

### Production of IFN-γ, TNF-α, and/or IL-17A by CD4^+^ T Cells After STM Stimulation

Main focus of the study was the analysis of a *Salmonella*-specific CD4^+^ T-cell response in regard to IFN-γ, TNF-α, and IL-17A production. To that end, PBMC as well as lymphocytes isolated from spleen, tonsils, jejunal lymph nodes, ileocolic lymph nodes, jejunum and ileum collected on final SDs were stimulated *in vitro* with either the vaccine strain or the challenge infection strain. Cells cultivated in medium-only served as negative controls. For analysis of cytokine-producing cells and their phenotype, multi-color FCM was performed. To identify *Salmonella*-specific CD4^+^ T cells, CD4^+^ cells were gated within live lymphocytes and further analyzed for IFN-γ, TNF-α and IL-17A production (**Figure S1A**). Subsequently, Boolean gating was applied, resulting in seven possible cytokine-producing phenotypes: IFN-γ single-producing, TNF-α single-producing, IL-17A single-producing, IFN-γ/TNF-α co-producing, IFN-γ/IL-17A co-producing, TNF-α/IL-17A co-producing and IFN-γ/TNF-α/IL-17A triple-producing cells. Raw frequencies of these cytokine-producing phenotypes for all organs and *in vitro* stimulations are listed in **Table S1**.

The frequencies of cytokine-producing CD4^+^ T cells varied between different animals and organs following stimulation with STM (**Figures S1B–D**). Comparing frequencies of cytokine-producing CD4^+^ T cells across organs, highest frequencies of *Salmonella*-specific CD4^+^ T cells for all cytokine-related phenotypes could be observed in LPL preparations of jejunum and ileum (**Figure S2**, N.B. the scaling of the y-axes in this figure varies between jejunum/ileum, lymphoid organs and blood/spleen).

To work out the significance of noted differences between control group and V+I animals, we chose to model the proportions of cytokine-producing CD4^+^ T cells for all phenotypes, animals and organs in a generalized linear mixed model. All samples from control animals as well as non-stimulated samples from V+I animals that were cultivated in medium-only served as reference values. The effect of each pig as an individual strongly influenced the data and therefore residuals were calculated for each sample to minimize the effect of the factor ‘animal’. Principal component analysis (PCA) and heat map analysis were carried out on residuals of cytokine-producing CD4^+^ T cells. With the exception of IFN-γ single-producing CD4^+^ T cells in blood, no significant differences in frequency of cytokine-producing CD4^+^ T cells were found between stimulation with the vaccine strain and the challenge infection strain (**Table S2**). Furthermore, no separate clustering could be observed between both stimulation variants in PCA and heat map analysis (data not shown). For these reasons, data from both stimulation variants were combined for all subsequent analyses.

### Cluster Analysis for STM-Stimulated IFN-γ/TNF-α/IL-17A Producing CD4^+^ T Cells

**Figure 3:**
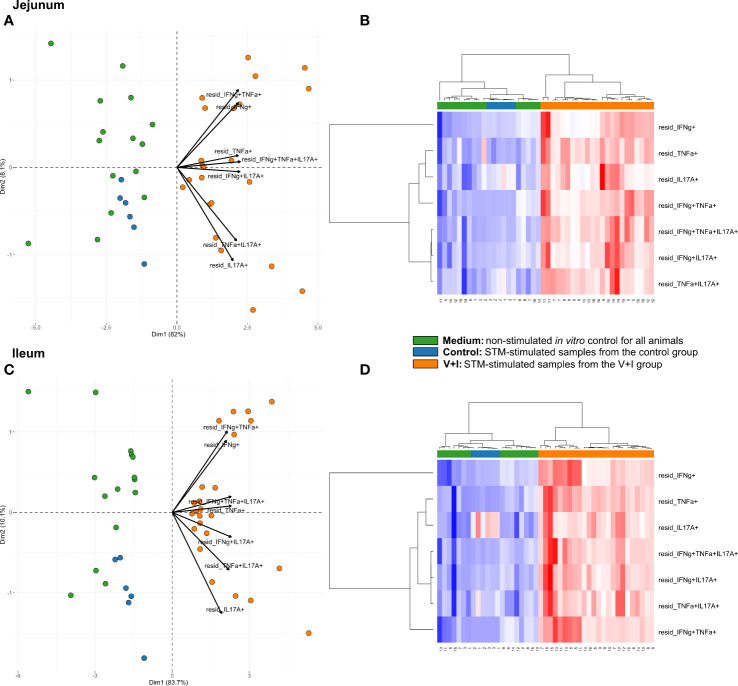
Unsupervised cluster analysis for *Salmonella* Typhimurium (STM)-stimulated interferon-γ (IFN-γ)/tumor necrosis factor-α (TNF-α)/interleukin-17A (IL-17A) producing CD4^+^ T cells isolated from jejunum and ileum. **(A)** Principal component analysis (PCA) based on residuals calculated from cytokine-producing CD4^+^ T cells derived from jejunum and stimulated *in vitro* with STM or medium-only. Each colored circle represents one of three samples derived from one animal. Samples stimulated with STM (vaccine or challenge strain) are depicted in orange for V+I animals and blue for control animals; samples from both groups cultivated in medium-only are shown in green. Arrows depict cytokine-producing phenotypes. **(B)** Heat map of residuals calculated from cytokine-producing CD4^+^ T cells derived from jejunum and stimulated *in vitro* with STM or medium-only. Heat maps, generated from data converted to Z-scores, show increase (red) or decrease (blue) of relative abundance of cytokine-producing CD4^+^ T cells. Each row represents a cytokine-producing phenotype and each column a sample. Numbers below columns indicate numbers of the individual animals. Samples stimulated with STM (vaccine or challenge strain) are indicated by orange boxes for V+I animals and blue boxes for control animals; samples from both groups cultivated in medium-only are indicated by green boxes. **(C, D)** Same as **(A)**, **(B)** but for cytokine-producing CD4^+^ T cells derived from the ileum.

STM primarily infects the gastrointestinal tract and does so by breaching the gut epithelial barrier. To examine the local T-cell immune response at this site of invasion, cluster analysis was first conducted for the two intestinal sections (jejunum, ileum, **Figure 3**). Dimension 1 of the PCA revealed a clear separation of the gut tissue-derived data points into two fractions: Samples from V+I animals stimulated with STM clustered together (orange dots) on one side. On the opposite side, all samples from control animals (blue dots if stimulated with STM antigen, green dots if cultivated in medium) as well as V+I samples cultivated in medium-only (remaining green dots) formed a cluster (**Figures 3A, C**). Hence, Dimension 1 resulted out of the animal treatment (control vs. vaccination and infection) as well as stimulation of the individual samples (medium vs. STM antigen). For both intestinal sections, Dimension 1 accounted for more than 80% of the variation. Dimension 2, which accounted for 8.1% and 10.1% of variation in jejunum and ileum, respectively, is associated with the type of cytokine production. Predominantly IFN-γ single- and IFN-γ/TNF-α co-producing cells clustered together in the upper quadrant while IL-17A containing phenotypes were grouped toward the other direction. Findings of the PCA were corroborated by results of the heat map analysis (**Figures 3B, D**). Heat maps show increases (red) and decreases (blue) of relative abundance of cytokine-producing CD4^+^ T cells. Relative abundances of phenotypes were markedly increased in samples from the V+I group that were stimulated with STM when comparing it to samples from control animals and medium-only samples. This resulted in two distinct clusters where STM-stimulated V+I samples are clearly separated from samples of control animals and samples from all animals that were cultivated in medium. Again similar to PCA results, hierarchical clustering of cytokine phenotypes in dendrograms showed that IL-17A^+^ phenotypes such as IFN-γ/TNF-α/IL-17A triple-producing, IFN-γ/IL-17A co-producing and TNF-α/IL-17A co-producing CD4^+^ T cells are closely related phenotypes while IFN-γ/TNF-α co-producing and IFN-γ single-producing CD4^+^ T cells clustered apart from them. To determine whether the observed differences between the control and the V+I group were significant, *p*-values were calculated for all phenotypes and organs and corrected for multiple testing where necessary (*p*-values below 0.1 were considered significant). Significant differences between control group and V+I group were found for 5 out of 7 phenotypes in both intestinal sections (**Table 1**). The phenotypes not reaching significant difference between groups were IL-17A single-producing CD4^+^ T cells in both intestinal tissues as well as IFN-γ single-producing CD4^+^ T cells in the jejunum and TNF-α/IL-17A co-producing CD4^+^ T cells in the ileum.

**Table 1:** Calculation of contrasts of *Salmonella*-specific CD4^+^ T-cell cytokine responses between control and V+I pigs in jejunum and ileum.

Tissue	Phenotype	Estimate*^a^*	SE*^b^*	t-ratio*^c^*	*p*-value*^d^*	fdr.p*^e^*
Jejunum	IFN-γ^+^	−1.942235	0.946641	−2.051713	0.052421	0.102649
	TNF-α^+^	−0.349227	0.109627	−3.185597	0.004483	0.048073
	IL-17A^+^	−0.194306	0.162531	−1.195501	0.244622	0.342471
	IFN-γ^+^TNF-α ^+^	−0.696878	0.262535	−2.654421	0.014868	0.051243
	IFN-γ^+^IL-17A^+^	−0.107349	0.045450	−2.361912	0.027440	0.074697
	TNF-α^+^IL-17A^+^	−0.087403	0.040816	−2.141406	0.043547	0.096991
	IFN-γ^+^TNF-α^+^IL-17A^+^	−0.116906	0.039699	−2.944789	0.007783	0.048073
Ileum	IFN-γ^+^	−1.722149	0.661531	−2.603277	0.016732	0.051243
	TNF-α^+^	−0.318299	0.105505	−3.016924	0.006867	0.048073
	IL-17A^+^	−0.030348	0.090255	−0.336242	0.739993	0.771482
	IFN-γ^+^TNF-α ^+^	−0.608607	0.231357	−2.630603	0.016001	0.051243
	IFN-γ^+^IL-17A^+^	−0.132379	0.045710	−2.896093	0.008366	0.048073
	TNF-α^+^IL-17A^+^	−0.079415	0.040456	−1.963030	0.062708	0.113803
	IFN-γ^+^TNF-α^+^IL-17A^+^	−0.144465	0.047416	−3.046742	0.005979	0.048073

a estimate: contrasts between least square means of control versus V+I pigs.

b SE: standard errors of the estimated contrasts.

c t-ratio: t test statistics.

d p-value: nominal p-values.

e fdr.p: p-values were corrected for multiple testing via false discovery rate. p-values < 0.1 were considered significant and are highlighted in gray.

**Figure 4:**
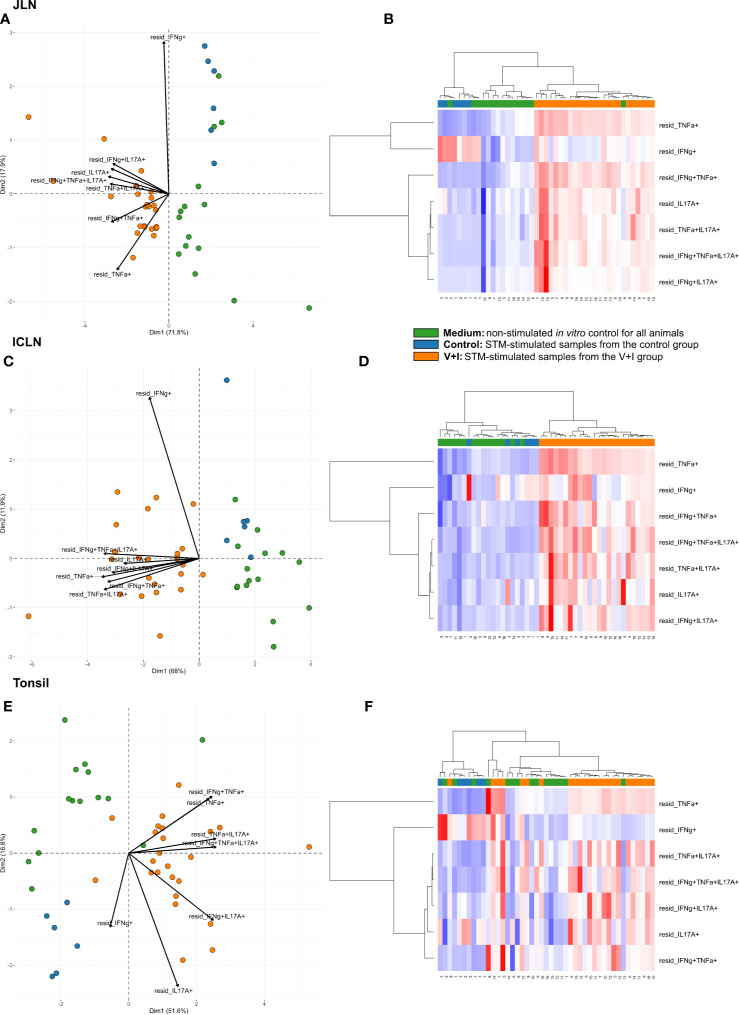
Unsupervised cluster analysis for *Salmonella* Typhimurium (STM)-stimulated interferon-γ (IFN-γ)/tumor necrosis factor-α (TNF-α)/interleukin-17A (IL-17A) producing CD4^+^ T cells isolated from jejunal lymph node (JLN), ileocolic lymph node (ICLN) and tonsil. **(A)** Principal component analysis (PCA) based on residuals calculated from cytokine-producing CD4^+^ T cells derived from jejunal lymph node and stimulated *in vitro* with STM or medium-only. Each colored circle represents one of three samples derived from one animal. Samples stimulated with STM (vaccine or challenge strain) are depicted in orange for V+I animals and blue for control animals; samples from both groups cultivated in medium-only are shown in green. Arrows depict cytokine-producing phenotypes. **(B)** Heat map of residuals calculated from cytokine-producing CD4^+^ T cells derived from jejunal lymph node and stimulated *in vitro* with STM or medium-only. Heat maps, generated from data converted to Z-scores, show increase (red) or decrease (blue) of relative abundance of cytokine-producing CD4^+^ T cells. Each row represents a cytokine-producing phenotype and each column a sample. Numbers below columns indicate numbers of the individual animals. Samples stimulated with STM (vaccine or challenge strain) are indicated by orange boxes for V+I animals and blue boxes for control animals; samples from both groups cultivated in medium-only are indicated by green boxes. **(C–F)** Same as **(A)**, **(B)** but for CD4^+^ T cells derived from ileocolic lymph node **(C, D)** and tonsil **(E, F)**.

Looking at the set of lymphatic organs, clustering in both intestinal lymph nodes was similar to that observed in jejunum and ileum, for both PCA and heat maps (**Figures 4A–D**). In both mesenteric lymph nodes, STM-stimulated V+I samples clustered away from control and medium-only samples with Dimension 1 accounting for approximately 70% of the variation (**Figures 4A, C**). Of note, IFN-γ single-producing CD4^+^ T cells clustered separately from all other phenotypes in the PCA. This is most probably due to an increase in abundance of cytokine-producing cells in some control animals for this phenotype as displayed in the corresponding heat maps (**Figures 4B, D**). Accordingly, differences in relative abundance of IFN-γ single-producers between groups did not reach significance for either lymph node (**Table 2**). Comparable to observations in the intestine, phenotypes that included IL-17A also formed closely related clusters in both lymph nodes as illustrated by heat map dendrograms. *P*-values for both lymph nodes revealed significant differences between control conditions and STM-stimulated samples from V+I animals for TNF-α single-producing, IFN-γ/TNF-α co-producing and TNF-α/IL-17A co-producing CD4^+^ T cells. Additional significant differences were reached for IFN-γ/IL-17A co-producing and IFN-γ/TNF-α/IL-17A triple-producing CD4^+^ T cells in ileocolic lymph nodes. In the tonsil, however, separation of STM-stimulated V+I samples from control animals and medium-only samples was less distinct (**Figures 4E, F**). Correspondingly, no significant differences in abundance of cytokine-producing phenotypes could be detected between groups in the tonsil (**Table 2**).

**Table 2:** Calculation of contrasts of *Salmonella*-specific CD4^+^ T-cell cytokine responses between control and V+I pigs in jejunal lymph node (JLN), ileocolic lymph node (ICLN) and tonsil.

Tissue	Phenotype	Estimate^1^	SE^2^	t-ratio^3^	*p*-value^4^	fdr.p^5^
JLN	IFN-γ^+^	0.067079	0.072485	0.925421	0.367107	0.473375
	TNF-α^+^	−0.127268	0.047231	−2.694616	0.014723	0.051243
	IL-17A^+^	−0.008054	0.010468	−0.769364	0.451359	0.552915
	IFN-γ^+^TNF-α ^+^	−0.048463	0.019869	−2.439113	0.024844	0.071611
	IFN-γ^+^IL-17A^+^	−0.004625	0.003549	−1.303352	0.205662	0.305376
	TNF-α^+^IL-17A^+^	−0.012488	0.005579	−2.238111	0.035507	0.084510
	IFN-γ^+^TNF-α^+^IL-17A^+^	−0.016620	0.008079	−2.057324	0.051583	0.102649
ICLN	IFN-γ^+^	0.005701	0.015205	0.374958	0.711656	0.758068
	TNF-α^+^	−0.057656	0.020898	−2.758968	0.012239	0.051243
	IL-17A^+^	−0.001802	0.001700	−1.060605	0.298201	0.394914
	IFN-γ^+^TNF-α ^+^	−0.013547	0.004851	−2.792814	0.010159	0.049781
	IFN-γ^+^IL-17A^+^	−0.001434	0.000554	−2.585914	0.015449	0.051243
	TNF-α^+^IL-17A^+^	−0.003867	0.001238	−3.124297	0.004408	0.048073
	IFN-γ^+^TNF-α^+^IL-17A^+^	−0.004843	0.001243	−3.897945	0.000705	0.034531
Tonsil	IFN-γ^+^	0.034242	0.020767	1.648844	0.113169	0.178880
	TNF-α^+^	−0.102940	0.050402	−2.042367	0.054467	0.102649
	IL-17A^+^	0.001278	0.005386	0.237207	0.814933	0.831910
	IFN-γ^+^TNF-α ^+^	−0.014022	0.007906	−1.773586	0.089954	0.157420
	IFN-γ^+^IL-17A^+^	−0.003559	0.002034	−1.749303	0.094375	0.159461
	TNF-α^+^IL-17A^+^	−0.004241	0.003743	−1.133177	0.272138	0.370410
	IFN-γ^+^TNF-α^+^IL-17A^+^	−0.004049	0.002521	−1.606028	0.124307	0.190346

^1^estimate: contrasts between least square means of control versus V+I pigs.

^2^SE: standard errors of the estimated contrasts.

^3^t-ratio: t test statistics.

^4^p-value: nominal p-values.

^5^fdr.p: p-values were corrected for multiple testing via false discovery rate. p-values < 0.1 were considered significant and are highlighted in gray.

**Figure 5:**
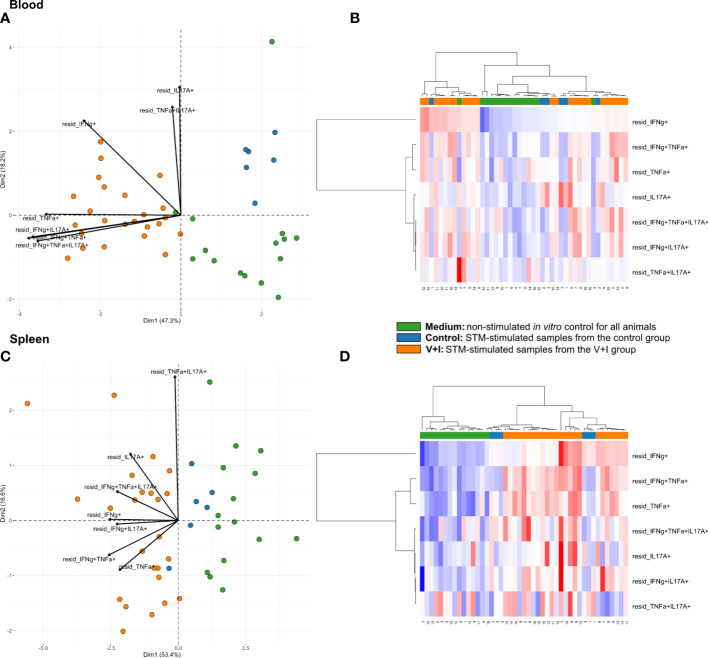
Unsupervised cluster analysis for *Salmonella* Typhimurium (STM)-stimulated interferon-γ (IFN-γ)/tumor necrosis factor-α (TNF-α)/interleukin-17A (IL-17A) producing CD4^+^ T cells isolated from blood and spleen. **(A)** Principal component analysis (PCA) based on residuals calculated from cytokine-producing CD4^+^ T cells derived from blood and stimulated *in vitro* with STM or medium-only. Each colored circle represents one of three samples derived from one animal. Samples stimulated with STM (vaccine or challenge strain) are depicted in orange for V+I animals and blue for control animals; samples from both groups cultivated in medium-only are shown in green. Arrows depict cytokine-producing phenotypes. **(B)** Heat map of residuals calculated from cytokine-producing CD4^+^ T cells derived from blood and stimulated *in vitro* with STM or medium-only. Heat maps, generated from data converted to Z-scores, show increase (red) or decrease (blue) of relative abundance of cytokine-producing CD4^+^ T cells. Each row represents a cytokine-producing phenotype and each column a sample. Numbers below columns indicate numbers of the individual animals. Samples stimulated with STM (vaccine or challenge strain) are indicated by orange boxes for V+I animals and blue boxes for control animals; samples from both groups cultivated in medium-only are indicated by green boxes. **(C, D)** Same as **(A)**, **(B)** but for cytokine-producing CD4^+^ T cells derived from the spleen.

Cluster analysis for STM-stimulated cytokine-producing CD4^+^ T cells was also performed for blood and spleen to gain more insight into the systemic CD4^+^ T-cell immune response against STM (**Figure 5**). With a few exceptions, control and medium-only samples largely clustered away from STM-stimulated V+I samples and Dimension 1 accounted for around 50% of the variation in blood and spleen (**Figures 5A, C**, respectively). In contrast to analyzed gut tissue and lymph nodes, relative abundances of cytokine-producing CD4^+^ T cells scattered across animal groups and the type of *in vitro* stimulation (medium versus STM antigen) (**Figures 5B, D**). Nonetheless, significant differences between control and V+I group could be detected for IFN-γ/TNF-α co-producing CD4^+^ T cells in blood and spleen, with additional significant differences for IFN-γ/IL-17A and IFN-γ/TNF-α/IL-17A co-producing CD4^+^ T cells in the blood (**Table 3**). Of note, IFN-γ/TNF-α co-producing CD4^+^ T cells constituted the only phenotype which was significantly different between control and V+I animals in all organs, with the exception of the tonsil.

**Table 3:** Calculation of contrasts of *Salmonella*-specific CD4^+^ T-cell cytokine responses between control and V+I pigs in blood and spleen.

Tissue	Phenotype	Estimate^1^	SE^2^	t-ratio^3^	*p*-value^4^	fdr.p^5^
Blood	IFN-γ^+^	−0.140874	0.310025	−0.454395	0.654487	0.712664
	TNF-α^+^	−0.018921	0.015141	−1.249649	0.225714	0.325293
	IL-17A^+^	0.014726	0.007054	2.087703	0.048858	0.102649
	IFN-γ^+^TNF-α ^+^	−0.090341	0.038238	−2.362602	0.029046	0.074908
	IFN-γ^+^IL-17A^+^	−0.004515	0.001471	−3.069605	0.005911	0.048073
	TNF-α^+^IL-17A^+^	−0.000276	0.000590	−0.467837	0.643481	0.712664
	IFN-γ^+^TNF-α^+^IL-17A^+^	−0.003946	0.001757	−2.246654	0.036219	0.084510
Spleen	IFN-γ^+^	−0.156988	0.264683	−0.593117	0.559227	0.651342
	TNF-α^+^	−0.032464	0.019558	−1.659894	0.111782	0.178880
	IL-17A^+^	−0.002837	0.004332	−0.654929	0.519200	0.620507
	IFN-γ^+^TNF-α ^+^	−0.129475	0.044655	−2.899429	0.008830	0.048073
	IFN-γ^+^IL-17A^+^	−0.002962	0.003282	−0.902414	0.376942	0.473594
	TNF-α^+^IL-17A^+^	0.000047	0.000795	0.059229	0.953292	0.953292
	IFN-γ^+^TNF-α^+^IL-17A^+^	−0.002789	0.004846	−0.575497	0.571586	0.651342

^1^estimate: contrasts between least square means of control versus V+I pigs.

^2^SE: standard errors of the estimated contrasts.

^3^t-ratio: t test statistics.

^4^p-value: nominal p-values.

^5^fdr.p: p-values were corrected for multiple testing via false discovery rate. p-values < 0.1 were considered significant and are highlighted in gray.

### Cluster Analysis of *Salmonella*-Specific Cytokine-Producing CD4^+^ T Cells Across Organs and Phenotypes

**Figure 6:**
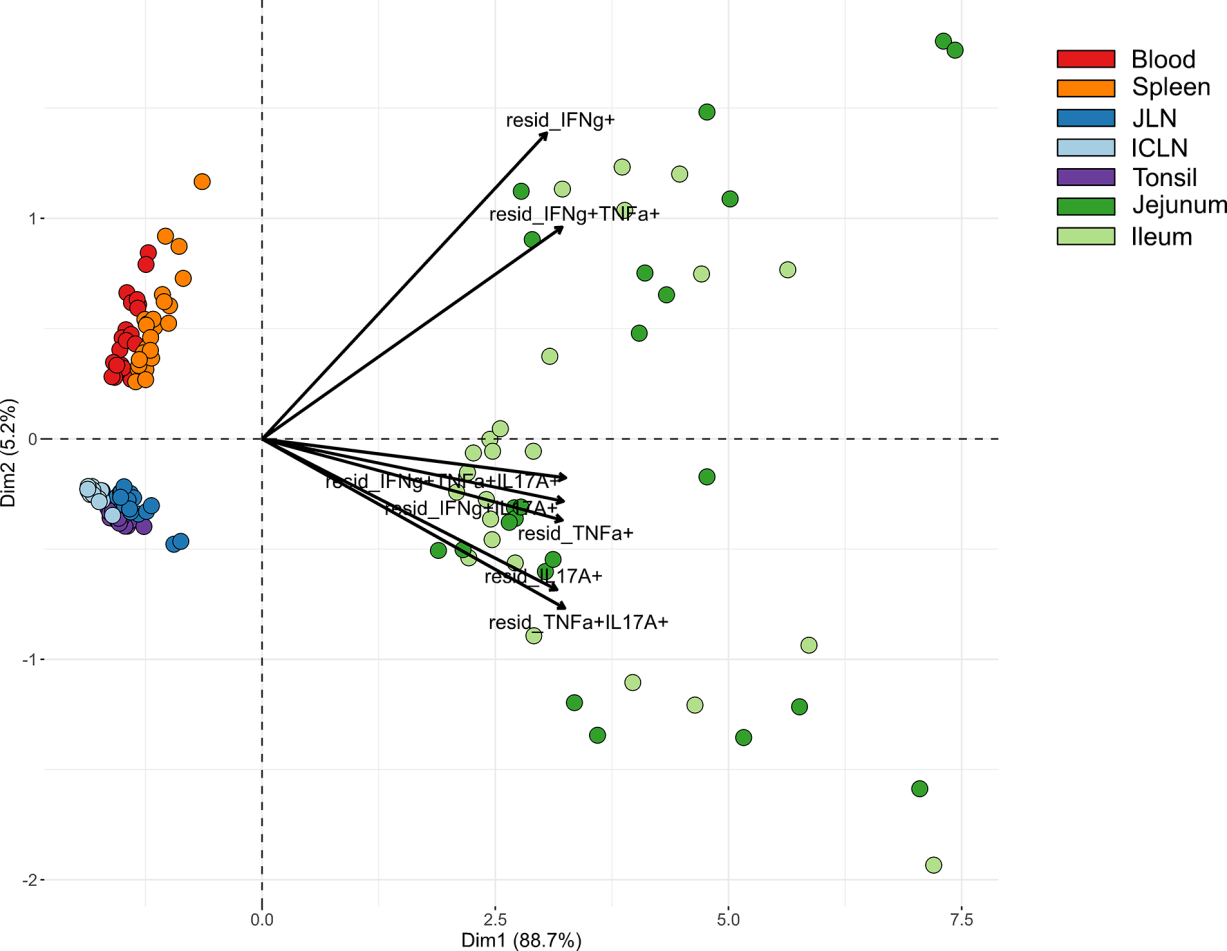
Principal component analysis (PCA) for STM-stimulated interferon-γ (IFN-γ)/tumor necrosis factor-α (TNF-α)/interleukin-17A (IL-17A) producing CD4^+^ T cells isolated from various organs. PCA based on residuals calculated from cytokine-producing CD4^+^ T cells derived from jejunum, ileum, jejunal lymph node (JLN), ileocolic lymph node (ICLN), tonsil, blood and spleen after *in vitro* stimulation with STM. Each colored circle represents one of two samples from one animal (stimulation with either vaccine or challenge strain). Samples are colored according to the organ of origin (blood: red, spleen: orange, JLN: blue, ICLN: light blue, tonsil: purple, jejunum: green, ileum: light green). Arrows depict cytokine-producing phenotypes.

Due to marked differences between organs in terms of both frequencies of cytokine-producing CD4^+^ T cells and the type of cytokine production, we carried out a cluster analysis for STM-stimulated samples from V+I animals that encompassed all seven organs. Here, the formation of three clusters by PCA could be observed (**Figure 6**). Blood and spleen as the two systemic organs formed one cluster with tonsil and lymph nodes forming another tight cluster of lymphatic origin on the same side, while all samples from the gut tissue clustered separately. As can be seen from the raw data (**Figure S2**), the abundances of *Salmonella*-specific cytokine-producing CD4^+^ T cells in jejunum and ileum highly surmounted those detected in the other organs as indicated by the arrows for all cytokine-producing phenotypes pointing toward the cluster of gut tissue samples (**Figure 6**). Accordingly, Dimension 1 accounted for 88.7% variability in the data set. Of note, Dimension 2 accounted only for 5.2% of the variability, but still gave a clear separation of blood and spleen versus the lymphatic organs. Combining this with the cytokine production phenotypes, where IFN-γ-single producers and IFN-γ/TNF-α co-producing cells separated from the other phenotypes, this indicates that IL-17A-producing phenotypes are less prominent in the systemic immune response found in blood and spleen.

Clustering among cytokine-producing phenotypes as seen in heat map dendrograms (**Figures 3**, **4**, and **5B, C**) also featured such a grouping. It could be observed across organs that IL-17A-containing phenotypes formed closely related clusters whereas IFN-γ single-, TNF-α single- and IFN-γ/TNF-α co-producing CD4^+^ T cells largely clustered separately. To study this from an alternate point of view, heat maps were calculated individually for all cytokine-producing phenotypes (**Figure S3**). For the majority of investigated phenotypes, distinct clusters emerged for STM-stimulated V+I samples on the one hand and control and medium-only samples on the other hand. Matching with observations from **Figure 6**, the dendrograms showed that organs predominantly form clusters according to their respective origins from systemic, lymphatic or gut tissue.

### Expression of CD8α and CD27 on *Salmonella*-Specific Cytokine-Producing CD4^+^ T Cells

**Figure 7:**
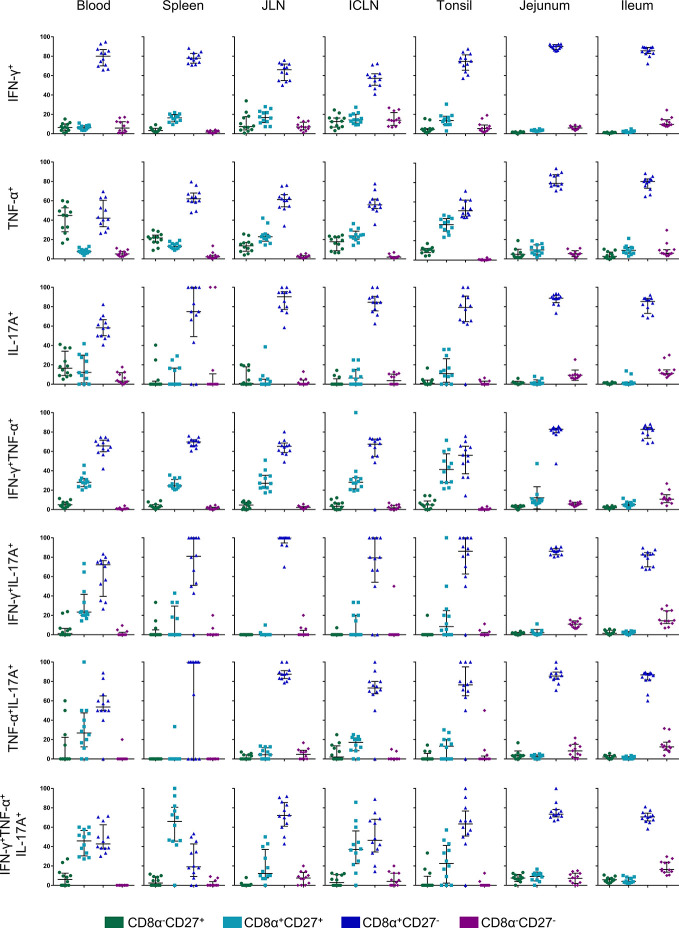
CD8α and CD27 expression of cytokine-producing CD4^+^ T cells in analyzed organs. Intracellular cytokine staining was performed on lymphocytes isolated from organ tissue following overnight *in vitro* stimulation with the challenge strain. CD4^+^ T cells were analyzed for expression of interferon-γ (IFN-γ), tumor necrosis factor-α (TNF-α), and interleukin-17A (IL-17A) and grouped into seven cytokine-producing phenotypes. Cytokine-producing CD4^+^ T cells were further subgated for CD8α and CD27 to identify four CD8a/CD27-defined cell populations: CD8α^-^CD27^+^ (green), CD8α^+^CD27^+^ (light blue), CD8α^+^CD27^-^ (dark blue), CD8α^-^CD27^-^ (violet). Individual graphs indicate percentages of CD8a/CD27-defined cell populations for each cytokine-producing phenotype from individual animals of the V+I group. Black bars indicate the median and whiskers show the interquartile range. Data was obtained from day of necropsy of the respective animals.

It has been shown previously that porcine CD4^+^ T cells can be differentiated into naïve CD4^+^ T cells with a CD8α^-^CD27^+^ phenotype, whereas CD8α^+^CD27^+^ and CD8α^+^CD27^-^ CD4^+^ T cells constitute central memory (Tcm) and effector memory (Tem) subsets, respectively (32). We therefore analyzed CD8α alongside CD27 expression for all seven cytokine-producing phenotypes in all organs (**Figure 7**). The gating strategy for the identification of these phenotypes is shown in **Figure S4**. Representative raw data for the CD8α/CD27 expression of cytokine-producing CD4^+^ T cells following STM-stimulation is shown in **Figure S5**. As illustrated in **Figure 7**, hardly any *Salmonella*-specific cytokine-producing CD4^+^ T cells with the hitherto uncharacterized CD8α^-^CD27^-^ phenotype were found. Also, CD8α^-^CD27^+^ naïve T cells showed only a low capacity for cytokine production with some exceptions such as for single TNF-α and IL-17A-producers in the blood. Instead, *Salmonella*-specific cytokine-producing CD4^+^ T cells predominantly had a CD8α^+^CD27^-^ effector memory phenotype, especially in the gut tissue. While a CD8α^+^CD27^+^ central memory population was present primarily for IFN-γ/TNF-α double- and IFN-γ/TNF-α/IL-17A triple-producing CD4^+^ T cells within analyzed non-gut locations, cytokine phenotypes present in jejunum and ileum were almost exclusively effector memory T cells.

## Discussion

Information on the porcine T-cell response against STM is still scarce. For the most part, previous studies have focused on detecting local and systemic immune responses on mRNA level and discovered that STM infection in the pig causes upregulation of mRNA expression of cytokines like IFN-γ, TNF-α, and IL-1β (16–20). However, the phenotype of the cells producing these mRNA transcripts was not investigated. Only one study also measured the expression of transcription factors T-bet, Gata-3 and Foxp3 in CD4^+^ T cells following STM infection (41) but the results were not fully conclusive. Differently, in the murine model CD4^+^ T cells are widely recognized as the main immune cell subset responsible for protection against STM (21, 22). Hence, we decided to lay the focus of our study on CD4^+^ T cells.

Considering also the lack of knowledge on the magnitude of the T-cell response in swine to STM, we decided to stimulate the immune system by a double vaccination with Salmoporc and subsequent challenge infection with a virulent STM strain. We chose this regimen because it was also used in the past to demonstrate the efficacy of the vaccine. These studies showed that, in comparison to two vaccinations or single infection, STM-specific IgG titers in blood were highest after this combination of two vaccinations and subsequent challenge infection (11). Hence, we assumed that this might also indicate an activation of CD4^+^ T cells at the systemic level. We further reasoned that this would probably coincide with activation of T cells in the gut mucosa, since the vaccine and the challenge infection were applied orally.

Of the seven organs analyzed in this study, the highest colonization with STM was found in the tonsils. This is not surprising as the tonsil is known to be invaded by various bacteria, with STM frequently establishing a persistent infection in this location (42, 43). Of note, the tonsil was the tissue with the lowest frequencies of cytokine-producing CD4^+^ T cells and no significant differences for any cytokine phenotype were found between V+I animals and the control conditions. One possible explanation might be the location of the pathogen within the organ. Unlike jejunum and ileum where STM invades the epithelial cells, it mainly resides extracellularly in porcine tonsils (44), thus making it more difficult for the immune system to attack the pathogen. Studies in recent years also present evidence that bacterial gene expression related to survival and persistence of *Salmonella* in the tonsil is vastly different from gene expression related to these mechanisms in the gut and gut-associated lymph nodes (45–47). Another factor may be the anti-inflammatory nature of the tonsil. In humans, tonsil-derived dendritic cells only induce a weak T-cell response to mucosally encountered pathogens but rather maintain immunotolerance (48). This observation fits with studies in the pig where the presence of *Actinobacillus pleuropneumoniae* in the tonsil induced an increase in IL-10 expression (49). Although IL-10 production was not measured in our study, a similar scenario is conceivable which could explain the poor induction of Th1 and Th17 responses in this organ.

Surpassing all other organs, we found the highest frequencies of cytokine-producing *Salmonella*-specific CD4^+^ T cells within LPLs isolated from jejunum and ileum. Of note, STM infection in the pig is mostly limited to the intestine, whereas a potential systemic infection is not well studied (50). Mouse models have shown that dissemination to mesenteric lymph nodes, blood and systemic organs can take place (51). We did not find *Salmonella* in spleen and liver of any of the animals two weeks post infection. As the immune system of V+I animals was already primed by vaccination prior to infection, it is possible that the infection was either already cleared or did not even spread to these systemic locations. In accordance to this, only few phenotypes of cytokine-producing CD4^+^ T cells showed significant rises in blood and spleen of V+I animals (**Table 3**). More phenotypes significantly different between V+I and control pigs were found for the investigated gut-draining lymph nodes (**Table 2**), whereas in the gut sections nearly all of the seven possible cytokine-producing phenotypes were induced (**Table 1**). This complies with studies in mice and humans, where T cells residing in the lamina propria have already been recognized as an important element of the response against STM and *Salmonella* Typhi (52–54). The presence of a very high abundance of *Salmonella*-specific cytokine-producing CD4^+^ T cells in the intestinal lamina propria of V+I animals in our study suggests that they also play a vital role in the mucosal immune response against STM infection in the pig.

Looking more closely at the seven possible cytokine-producing phenotypes in the different organs, it became apparent that IL-17A-containing phenotypes formed closely related clusters, often separating from IFN-γ and IFN-γ/TNF-α co-producing CD4^+^ T cells. This separation is especially striking in jejunum and ileum. Th17 cells and their signature cytokine IL-17A are well known for contributing to mucosal immunity and protection against intracellular enteric pathogens (55–57). Infection with STM has previously been shown to induce the expression of Th17 cytokines in the intestinal mucosa of other species such as mice, calves and rhesus macaques (27, 58). A depletion of Th17 cells in the intestinal mucosa of rhesus macaques by infection with simian immunodeficiency virus was correlated with increased STM dissemination to mesenteric lymph nodes (27) suggesting that Th17 cells are important in disease containment. On the other hand, it has been discussed that IL-17A-induced recruitment of neutrophils and the resulting inflammation is exploited by pathogens like STM and ultimately promotes bacterial colonization (59). Since all V+I animals were vaccinated before infection and we did not detect any signs of inflammation in their intestines, a negative impact of the *Salmonella*-specific Th17 phenotypes observed in this study seems unlikely. Instead, we provide evidence that Th17 cells also seem to play a protective role in host defense against STM in swine.

In contrast to other IL-17A^+^ populations, however, IL-17A single-producing CD4^+^ T cells were the only cytokine phenotype that did not reach significant difference between control and V+I pigs in any of the organs. Indeed, we observed across organs that CD4^+^ T-cell phenotypes consisting of more than one cytokine, such as IFN-γ/TNF-α, IFN-γ/IL-17A, TNF-α/IL-17A and IFN-γ/TNF-α/IL-17A, overall reached significant differences over the control group more frequently than single-cytokine producing CD4^+^ T cells. T cells producing several cytokines, also called multifunctional (MF) T cells, have been associated with protection in several bacterial and viral infections in humans and mice (60–63) and were found to be functionally superior to their single-producing counterparts (64). Studies in swine have also demonstrated the involvement of antigen-specific MF CD4^+^ T cells in response to various pathogens (65–68). While co-production of IFN-γ, TNF-α and IL-2 by T cells is frequently investigated, reports on these cytokines in combination with IL-17A in the context of infectious diseases are rather scarce. Concerning *Salmonella*, MF IL-17A T-cell responses have been reported in the blood and terminal ileum of humans. Vaccination or infection with *Salmonella* Typhi led to the induction of MF CD8^+^ and CD4^+^ T-cell responses, that, in case of MF CD8^+^ T cells in PBMC, were demonstrated to correlate with disease outcome (54, 69, 70). To our knowledge, this is the first description of simultaneous production of IL-17A with IFN-γ and TNF-α by antigen-specific CD4^+^ T cells in the pig and it can be speculated that MF CD4^+^ T cells may serve as a correlate of protection for STM infection in swine. Especially IFN-γ/TNF-α co-producing CD4^+^ T cells appear as a promising candidate as significant differences between groups were reached for six out of the seven analyzed organs for this phenotype, including the blood. Moreover, based on observations in the mouse model, it is likely that these cells contribute to STM clearance by a potent stimulation of macrophages that have engulfed the pathogen (23, 26).

Interestingly, it has recently been shown that non-cognate stimulation of Th1 cells contributes to resolution of *Salmonella* infection in mice (71, 72). This describes a mechanism where T cells are stimulated by T-cell receptor (TCR)-independent stimuli such as inflammatory cytokines without TCR recognition of cognate antigen presented by antigen presenting cells. Since we analyzed total CD4^+^ T cells in our study and used whole bacterial antigen for *in vitro* re-stimulation, it is possible that a fraction of the measured cytokine production by CD4^+^ T cells may be derived from non-cognate stimulation and not *via* direct stimulation of the TCR. So far, these bystander responses have mostly been described in effector or memory CD4^+^ T cells (73, 74). Considering the strong stimulation applied in our study with two vaccinations and a challenge infection, it is conceivable that some of the T cells in our analyses had already reached a differentiation status that may have enabled them to respond in a non-cognate manner. Although that could lead to slightly overestimated numbers of STM-specific CD4^+^ T cells in V+I animals, it might more accurately reflect the situation occurring *in vivo*.

For investigation of further functional differentiation, we looked at the expression of CD8α and CD27, which have been proposed for the distinction between central (Tcm) and effector memory (Tem) CD4^+^ T cells in the pig (32). *Salmonella*-specific cytokine-producing CD4^+^ T cells in all organs predominantly expressed CD8α while lacking CD27, corresponding to a Tem phenotype in the pig. Conversely, previous studies on viral infections in swine have shown that the CD4^+^ Tcm subset was capable of IFN-γ, TNF-α and/or IL-2 production (66, 75). However, we did detect two phenotypes, namely IFN-γ/TNF-α co-producing along with IFN-γ/TNF-α/IL-17A triple-producing CD4^+^ T cells, with a sizable cell population co-expressing CD8α and CD27 in non-gut tissues, indicating a Tcm subset. As IFN-γ/TNF-α co-producing CD4^+^ T cells were also present in significantly higher amounts in V+I compared to control animals in the blood, they might be exploited as a phenotype for STM T-cell immunity in the pig.

In jejunum and ileum, almost the entirety of all cells regardless of the cytokine phenotype displayed features of Tem cells. Indeed, it is mainly Tem cells that reach non-lymphoid tissues like the intestine (76, 77). The mucosal memory pool contains recirculating as well as resident memory T cells (Trm) that stay in the tissue long term to initiate an immediate immune response against enteric pathogens (77, 78). In humans, *Salmonella* Typhi-specific CD4^+^ Trm cells were induced in the ileal mucosa after Ty21a immunization (54). Moreover, resident memory Th1 cells have been shown to be indispensable for protection against STM infection in mice (30). Unfortunately, due to a lack of available antibodies for markers of tissue-residency, Trm cells cannot yet be identified in the pig. Nevertheless, we can speculate that a part of the population now identified as Tem cells in the gut may represent Trm cells. These cells may have been already induced by the vaccination, settled down permanently in the intestine and were re-activated upon challenge infection.

In conclusion, by vaccinating piglets with a live attenuated STM vaccine and challenging them with a virulent STM strain, we could show the induction of STM-specific multifunctional CD4^+^ T cells across organs with a strong enrichment in the intestinal mucosa. These cells predominantly possessed an effector memory phenotype. Their multifunctional cytokine profile suggests an involvement in protective immunity against STM infection and IFN-γ/TNF-α co-producing CD4^+^ T cells in the blood might be investigated further as a marker for long-term protective immunity against STM infections. Finally, we think that our study forms an important foundation for more in-depth studies on the T-cell response in pigs against STM following only vaccination or infection. In this way, the postulated correlates of protection will be further corroborated.

## Data Availability Statement

The raw data supporting the conclusions of this article will be made available by the authors, without undue reservation.

## Ethics Statement

The animal study was reviewed and approved by Advisory Committee for Animal Experiments, University of Veterinary Medicine, Vienna and the Federal Ministry for Science, Research and Economy (BMWF-68.205/0241-WF/V/3b/2016).

## Author Contributions

SSp, TT, VF, AS, and WG conceived the idea and designed the project. ES, CK, and AL organized the animal experiment and necropsy with sample collection. EV, AP, JL, KM, and MS performed lymphocyte isolation and *in vitro* stimulation. SSc performed flow cytometry experiments. JS conducted bacteriological analysis. MD performed statistical analysis. SSc and WG analyzed the experiments, interpreted the data, and wrote the manuscript. SSp, TT, VF, AL, and AS assisted with interpretation of the data. All authors contributed to the article and approved the submitted version.

## Funding

This project was funded by Ceva Innovation Center GmbH (formerly belonging to IDT Biologika GmbH), Dessau-Roßlau, Germany.

## Conflict of Interest

SSp, TT, and VF are employed by Ceva Innovation Center GmbH.

The authors declare that this study received funding from Ceva Innovation Center GmbH (formerly belonging to IDT Biologika GmbH), Dessau-Roßlau, Germany. The funder had the following involvement in the study: The co-authors employed by the funder were involved in the study design and interpretation of the results as indicated in the “author contribution” statement. However, this did not influence the scientific integrity of the study and the presented findings.

The remaining authors declare that the research was conducted in the absence of any commercial or financial relationships that could be construed as a potential conflict of interest.
