# Supplementary material for: Vaccination and Infection of Swine With Salmonella Typhimurium Induces a Systemic and Local Multifunctional CD4+ T-Cell Response
Source: Front Immunol. 2021 Jan 11;11:603089. doi: 10.3389/fimmu.2020.603089 (PMC7874209; doi:10.3389/fimmu.2020.603089)
Supplement: Supplementary file 1 [file DataSheet_1.docx]

Supplementary Material


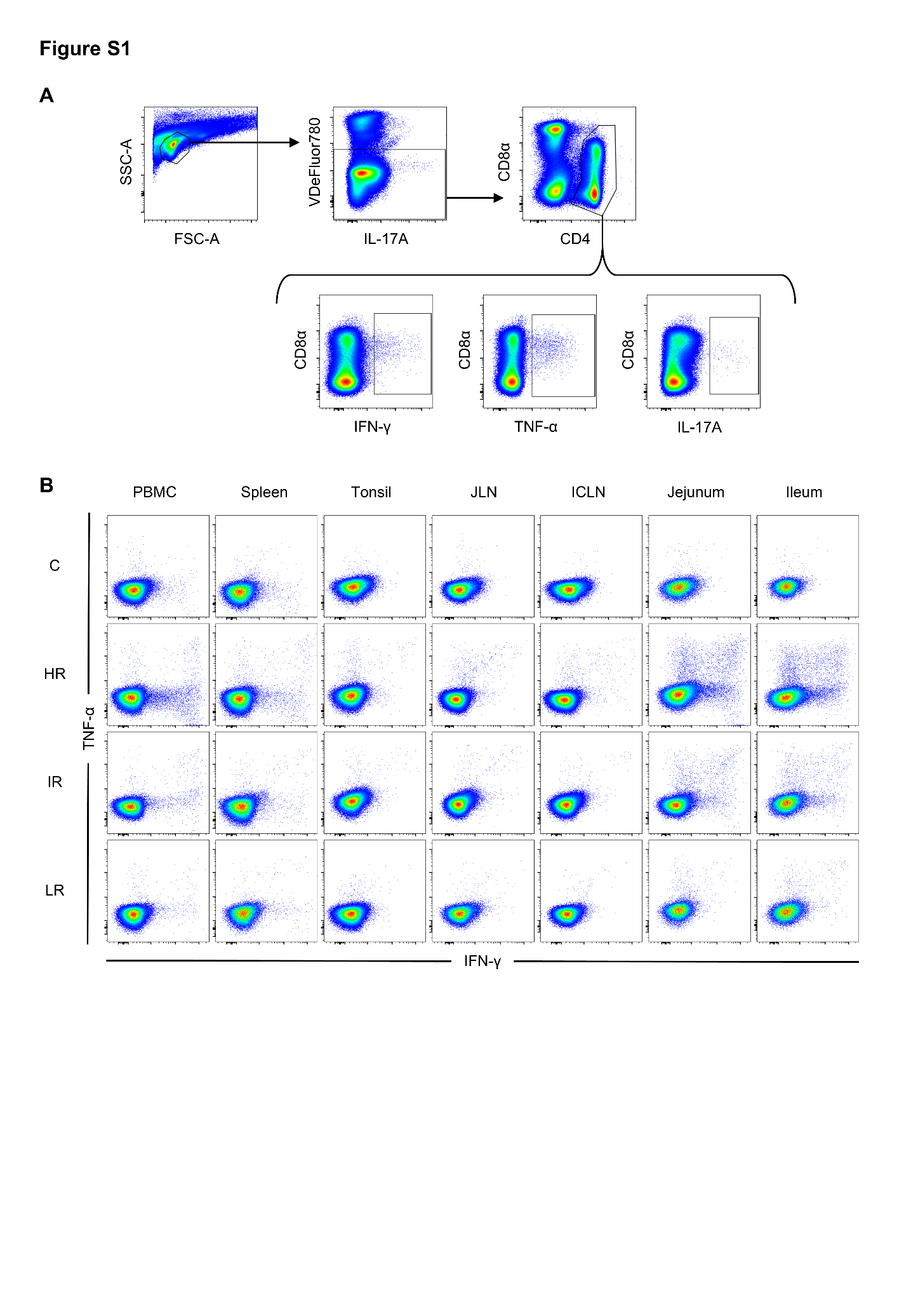


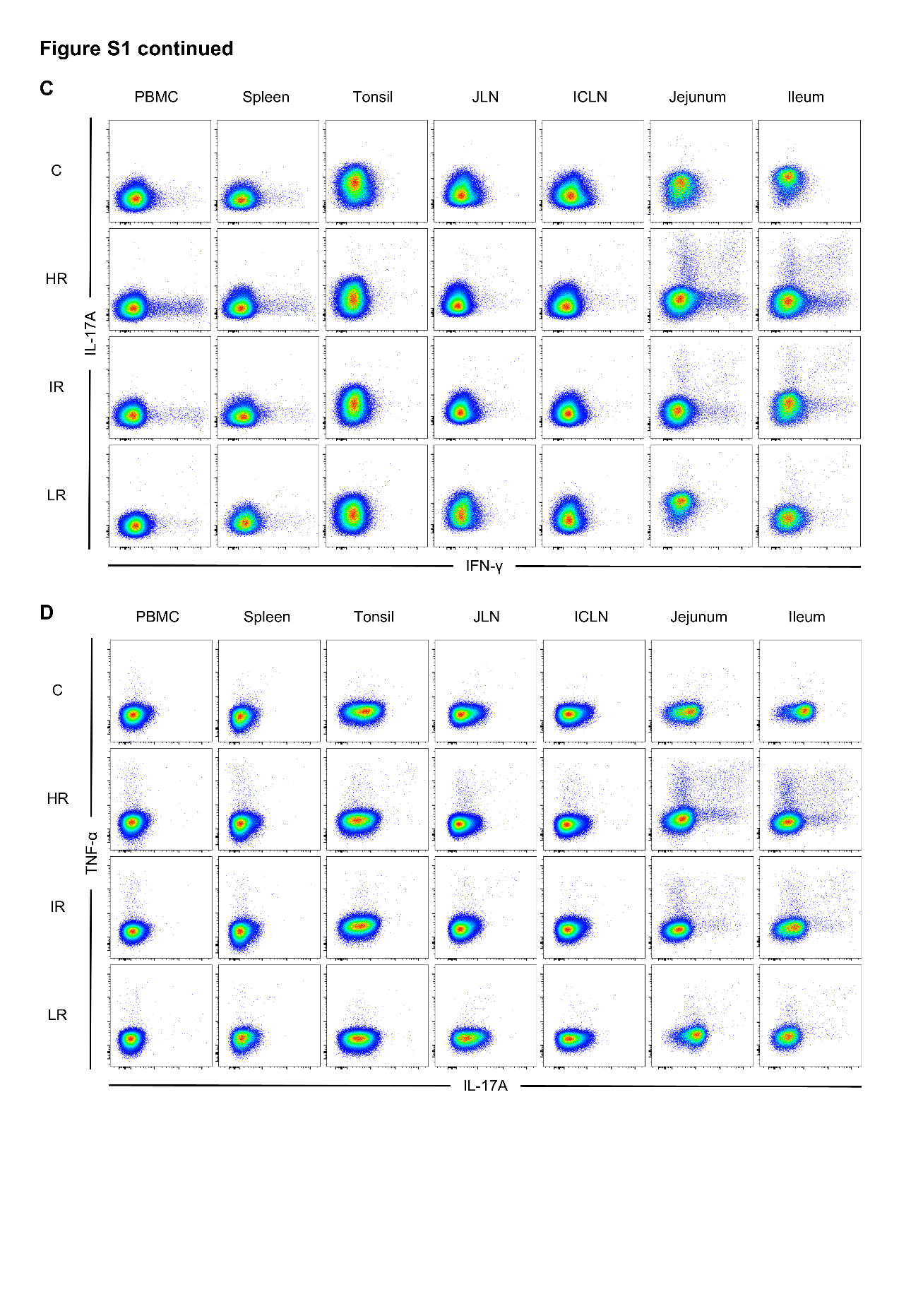


Figure S1. Gating strategy and representative raw data for FCM analysis of CD4^+^ T cells. Intracellular cytokine staining was performed on lymphocytes isolated from various locations following overnight *in vitro* stimulation with STM antigen. (A) Representative gating strategy for the intracellular cytokine staining in CD4^+^ T cells is shown (Sw #9, jejunal lymph node, stimulation with the vaccine strain). Cells were gated according to their light scatter properties and further subgated for live cells (VDeFlour780 negative). Subsequently, a gate was set on CD4^+^ cells and a Boolean gating for IFN-γ, TNF-α and IL-17A was performed. (B-D) Co-production of IFN-γ/TNF-α (B), IFN-γ/L-17A (C) and TNF-α/IL-17A (D) in CD4^+^ T cells in analyzed organs. Representative data from one high responder (HR), one intermediate responder (IR) and one low responder (LR) is shown. Additionally, one representative control (C) animal is shown for each organ.


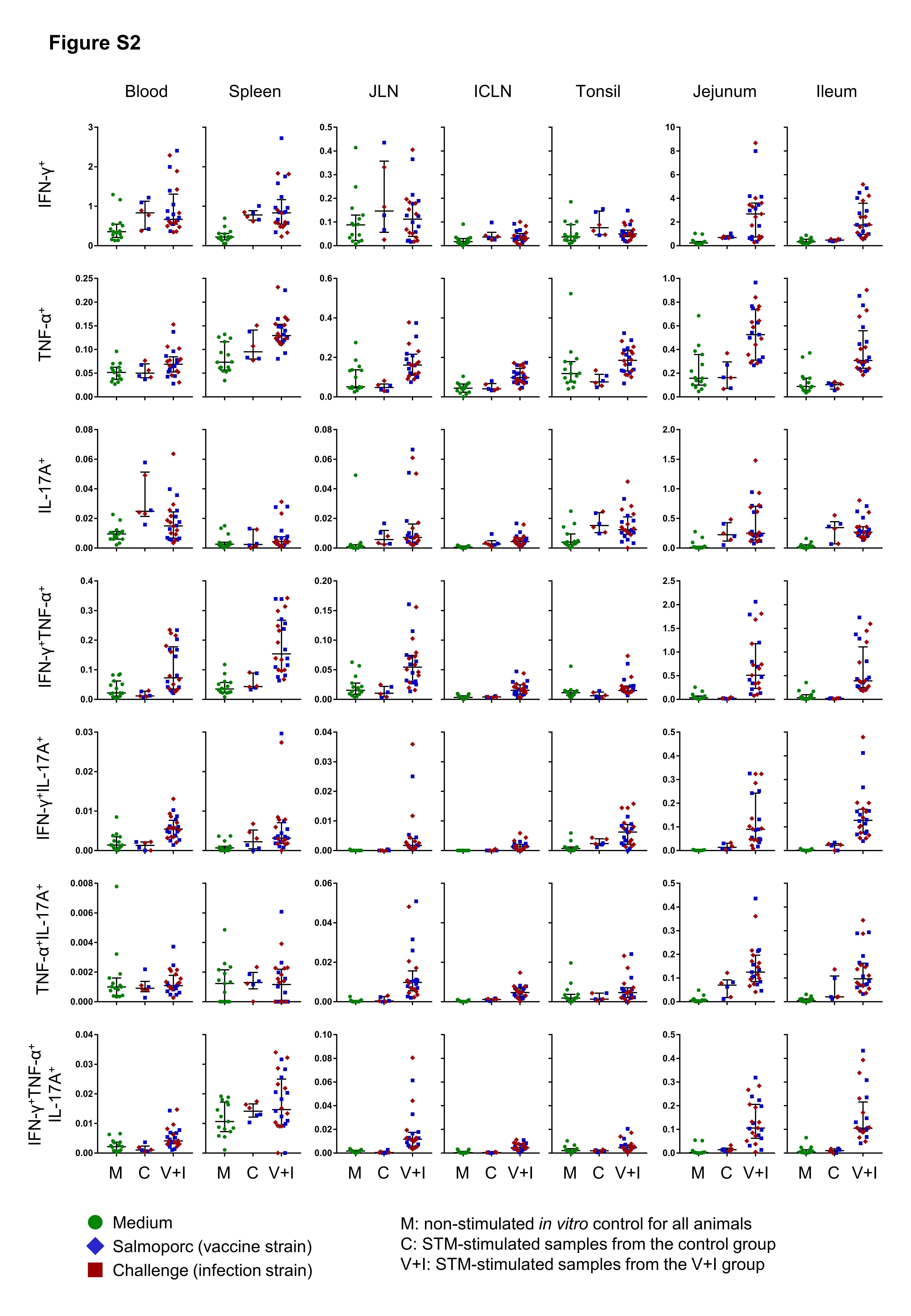


**Figure S2.** Frequencies of cytokine-producing CD4^+^ T cells in blood, spleen, tonsil, jejunal lymph node (JLN), ileocolic lymph node (ICLN), jejunum and ileum. CD4^+^ T cells were gated within live lymphocytes and further analyzed for IFN-γ, TNF-α and IL-17A production by Boolean gating. Individual graphs indicate percentages of cytokine-producing CD4^+^ T cells from individual animals of the control (C) and the V+I group (V+I) within total CD4^+^ T cells. Cells were stimulated with the vaccine strain (Salmoporc, blue diamonds) or the challenge infection strain (Challenge, red squares) or cultivated in medium-only (Medium, green circles). Samples from both groups in medium-only (M) are displayed on the left side of each graph. Black bars indicate the median and whiskers show the interquartile range. Data was obtained from the day of necropsy of the respective animals.

**Figure S3.** Heat map analysis for STM*-*stimulated IFN-γ/TNF-α/IL-17A producing CD4^+^ T cells isolated from various organs. Heat map of residuals calculated from cytokine-producing CD4^+^ T cells derived from jejunum, ileum, jejunal lymph node (JLN), ileocolic lymph node (ICLN), tonsil, blood and spleen stimulated *in vitro* with STM or medium-only. Each row represents an organ and each column a sample. Numbers under each column indicate animal numbers. Samples stimulated with STM (vaccine or challenge strain) are indicated by orange boxes for V+I animals and blue boxes for control animals; samples from both groups cultivated in medium-only are indicated by green boxes. Heat maps were generated from data converted to Z-scores. Relative increase (red) or decrease (blue) of abundance of cytokine-producing CD4^+^ T cells is shown.


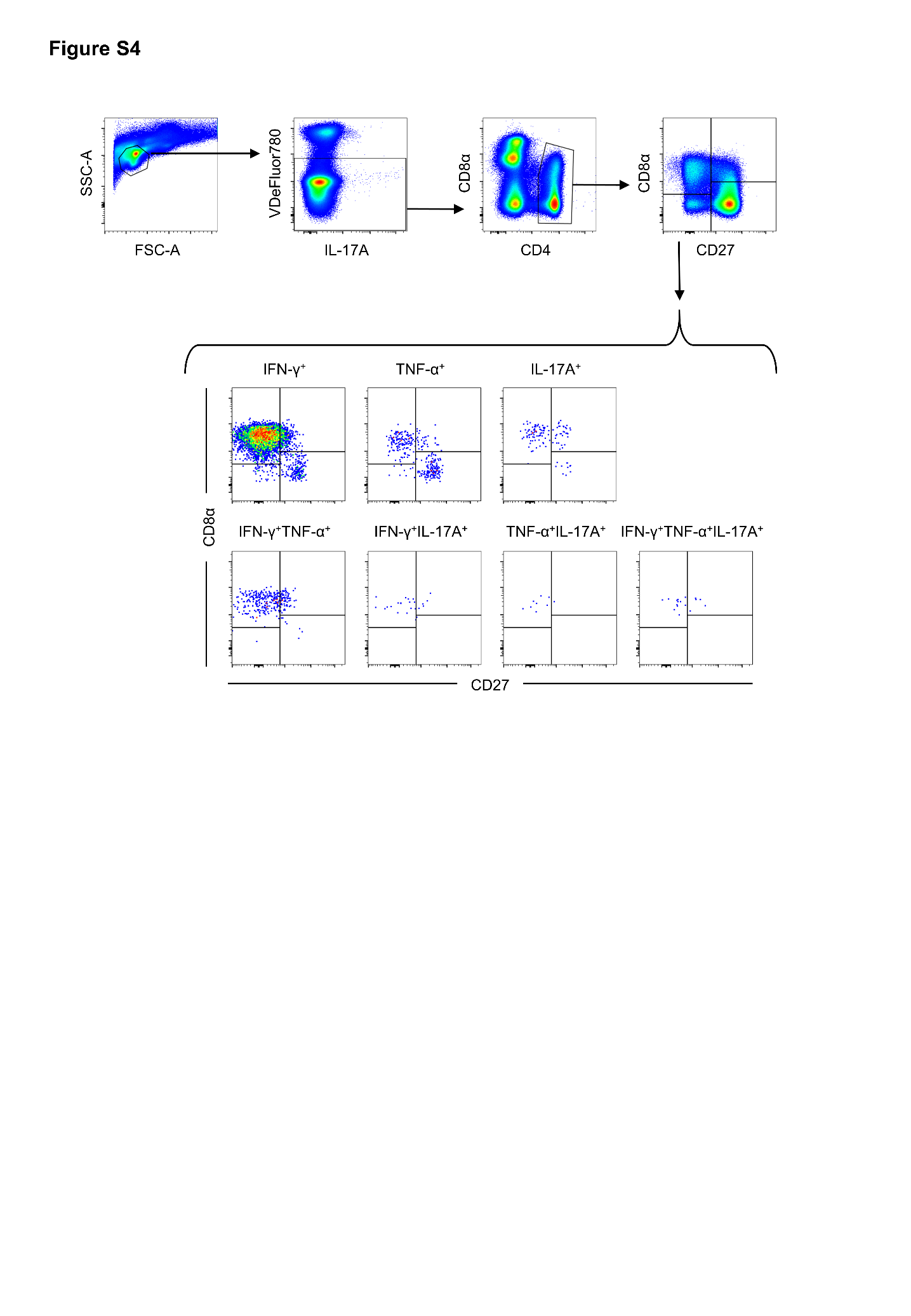
**Figure S4.** Gating strategy for identification of phenotypes based on CD8α/CD27 expression of cytokine-producing CD4^+^ T cells. Blood and organ-derived T cells were analyzed for CD8α/CD27-defined phenotypes within cytokine-producing CD4^+^ T cells. Representative data depicting gates used to identify CD8α/CD27-defined phenotypes from cytokine-producing CD4^+^ T cells of one pig is shown in pseudo-color plots. Dots were enlarged in plots of cytokine-producing phenotypes for better visibility of rare populations.

**
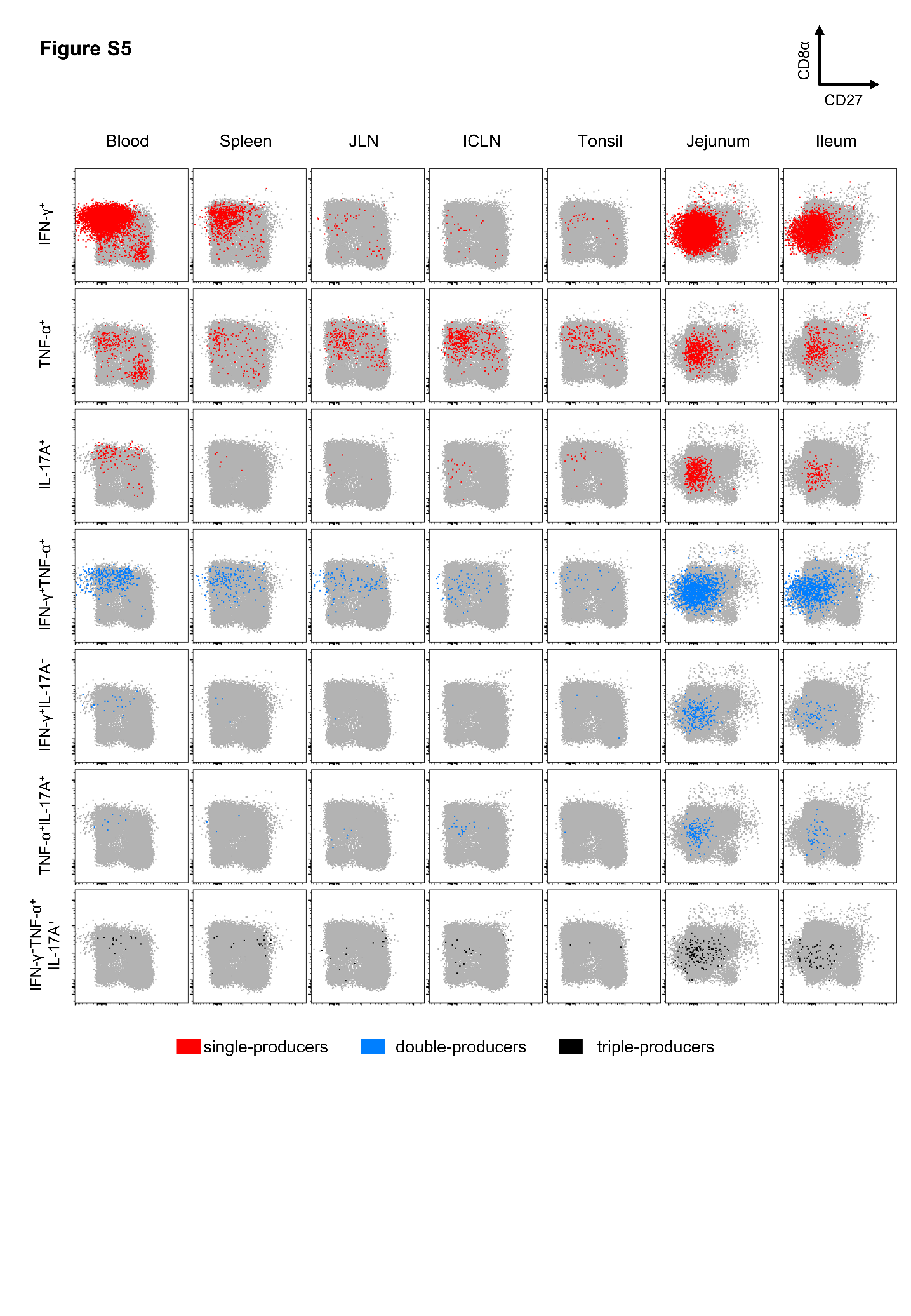
Figure S5.** CD8α and CD27 expression of cytokine-producing CD4^+^ T cells in analyzed organs. CD8α (y-axis) and CD27 expression (x-axis) was analyzed in total CD4^+^ T cells (light grey dots, background) and CD4^+^ T cells producing a single cytokine (red dots, top), two cytokines (blue dots, middle) or three cytokines (black dots, bottom). Raw data of one representative animal (Sw #11) is shown. Approximate numbers of CD4^+^ T cells depicted per organ and cytokine-producing phenotype: Blood: 4.2 x 10^5^, Spleen: 1.3 x 10^5^, JLN: 2.6 x 10^5^, ICLN: 2.8 x 10^5^, Tonsil: 1.7 x 10^5^, Jejunum: 7.3 x 10^4^, Ileum: 7.4 x 10^4^.
